# Medical Education during the COVID-19: A Review of Guidelines and Policies Adapted during the 2020 Pandemic

**DOI:** 10.3390/healthcare11060867

**Published:** 2023-03-16

**Authors:** Soichiro Saeki, Reiko Okada, Peter Y. Shane

**Affiliations:** 1 Faculty of Medicine, School of Medicine, Osaka University, Osaka 565-0871, Japan; 2 Center Hospital of the National Center for Global Health and Medicine, Tokyo 162-8655, Japan; 3 Osaka Police Hospital, Osaka 543-0035, Japan; 4 International Medical Department, Hokkaido University Hospital, Hokkaido 060-8648, Japan

**Keywords:** COVID-19, medical education, clinical clerkship, medical students

## Abstract

The novel coronavirus disease (COVID-19) pandemic has dramatically changed education systems as most governments around the world closed schools to prevent outbreaks on campus. Medical education was not immune from these policies, and medical students were deprived of opportunities, particularly in clinical training. To determine how countries worldwide have responded to the pandemic, we conducted a literature review of the policies and guidelines of four countries: Japan, the United States (USA), the United Kingdom (UK) and Australia, as well as case reports of faculty and medical students up to September, 2020. Although the methods of implementation were unique to each country, the concept of “returning medical students to live education as quickly and safely as possible” was common. However, the extent to which students and faculty members became engaged in the treatment process of COVID-19 varied. While some countries endorsed students to work as members of medical staff to treat COVID-19, other countries took measures to ensure the safety of both medical students and patients. We await further reports worldwide in order to better understand the strategies employed by different nations in preparation for future possible infection outbreaks.

## 1. Introduction

The coronavirus 2019 (COVID-19) outbreak has disrupted social systems worldwide, and medical education is not an exception. In order to prevent outbreaks occurring in and from medical schools, many students were forced to attend lectures or even clinical clerkships off-site [1,2,3], as hospitals only allowed in essential healthcare workers [4,5]. This led to a direct lack of educational opportunities for medical students, raising concerns about compromised quality. As medical education is designed to improve the quality of healthcare in society [6], this problem may have serious consequences for long-term public health. 

To the best of our knowledge, there has been no occurrence of such events that forced the medical educational system to change in such a drastic, intrinsic manner globally in the 21st century. The best way to tackle such hardship is to obtain the know-hows of other medical schools. Hence, comparing the medical education systems of several countries worldwide, along with some examples of how the faculty and students tried to cope with the situation, is of considerable importance. 

Previously, Fukushima published a study on the changes in medical education in the United States (USA) and the United Kingdom (UK) [7] as well as Japan [8] up to mid-May 2020. From the beginning, Japan’s policy has been to protect student safety by postponing or replacing clinical training with online or on-campus training, while the UK and the USA have encouraged medical students to take the initiative and participate in medical practice. Additionally, as of May, all of these guidelines were in the early planning stages, and to our knowledge, there is no comprehensive record of the subsequent guidelines for medical education in each country. Our main objective herein is to provide a summary of the above period and to update the policies of each country up to August and September, when the new academic year begins in the countries other than Japan. Additionally, we have extended the scope of our review to include policies from Australia, in order to gain a broader understanding of the changes in education as a result of the global COVID-19 outbreak.

## 2. Materials and Methods

In order to discern the modifications to the medical education system post-pandemic, we conducted a comprehensive literature review. The scope of our review was to analyze documented guidelines from institutional bodies as a subjective, overarching principle for each country, and to collect reports from faculties and students to provide a narrative perspective on the implementation of these guidelines. The countries from which we obtained information were Japan, the UK and the USA, which had previously been studied by Fukushima and Australia, which prior to COVID-19 had similar levels of medical resources and easy access to English-language information, as well as a difference in how the country dealt with the COVID-19 pandemic. As most of the information on the internet was updated according to the COVID-19 situation, we mainly obtained and compiled data from the websites of the respective institutions and universities as of December 2020 (re-searched on 16 March 2022). 

### 2.1. Literature Review

Regarding the global examination of medical educational policies, limited research has been conducted to evaluate the efficacy of such systems. Our primary source of information was not gleaned from any journal database, but rather obtained through official institutions, online media and other relevant sources via internet search engines. In particular, PubMed, Google Scholar and Web of Science were searched by two of the authors to cull information from academic references. Conversely, narrative insights from faculty and medical students were sourced from journal databases, including perspectives and written accounts from both researchers and students themselves.

### 2.2. Case Report

With regard to the adaptations made by universities in response to the COVID-19 pandemic, we surveyed official statements issued by universities regarding modifications to their curricula and sought out case studies that exemplified the experiences of both students and faculty. Additional information was gathered from the official websites of medical schools in the countries under examination. Due to a scarcity of resources providing narrative perspectives from students, the authors compiled a new case report based on their personal experiences at Osaka University Medical School in Japan, drawing on both official notifications provided to students by faculty administration and the authors’ own observations.

## 3. Results

**Figure 1:**
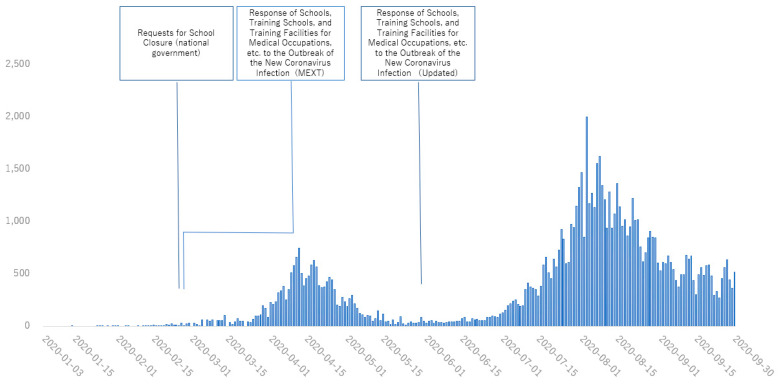
Policies for medical education from January to August, 2020 in Japan. Information was distributed from governments and other institutions in charge of medical education. (MEXT: Ministry of Education, Culture, Sports, Science and Technology).

**Figure 2:**
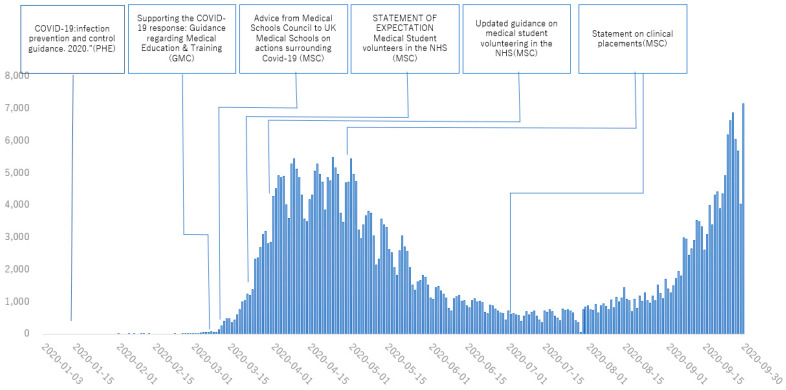
Policies for medical education from January to August, 2020 in United Kingdom. Information was distributed from governments and other institutions in charge of medical education. (PHE: Public Health England; GMC: General Medical Council; MSC: Medical Schools Council).

**Figure 3:**
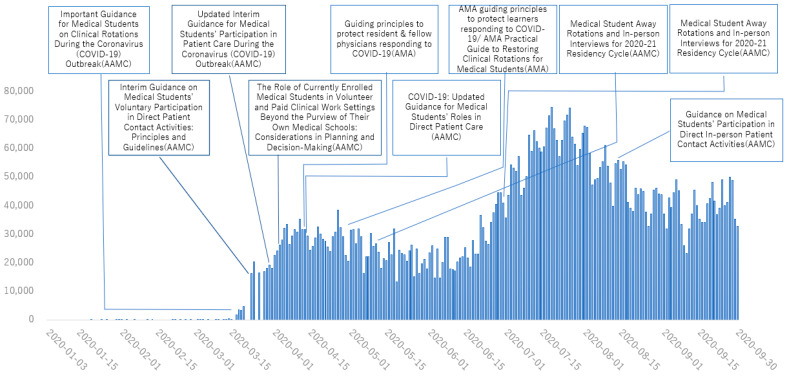
Policies for medical education from January to August, 2020 in United States. Information was distributed from governments and other institutions in charge of medical education. (AAMC: Association of American Medical Colleges; AMA: American Medical Association).

**Figure 4:**
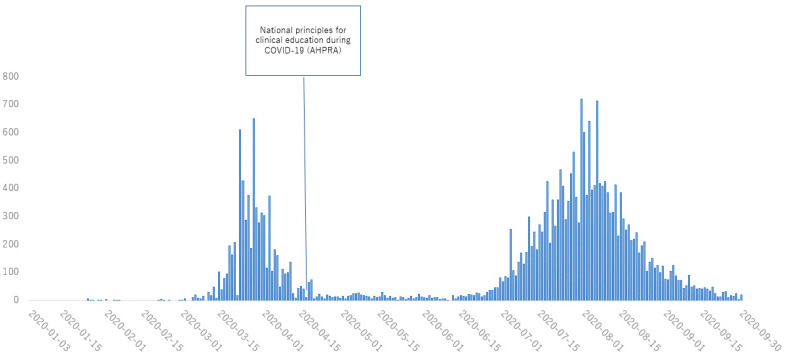
Policies for medical education from January to August, 2020 in Australia. Information was distributed from governments and other institutions in charge of medical education. (AHPRA: Australian Health Practitioner Regulation Agency).

Figure 1, Figure 2, Figure 3 and Figure 4 show the main policies along with the number of cases of COVID-19 in 2020 in Japan (Figure 1), the UK (Figure 2), the USA (Figure 3) and Australia (Figure 4).

### 3.1. Japan

#### 3.1.1. National Policies

In the wake of the initial outbreak of COVID-19, the Japanese government implemented a policy on February 27 mandating the closure of all primary, secondary and high schools [9] until the end of spring vacation, typically falling in the first week of April. However, the government declared a state of emergency on 7 April in seven prefectures, subsequently extending it to encompass all prefectures on 16 April, leading to prolonged school closures until mid-May [10]. Universities were also compelled to suspend on-campus lectures. In response, the Ministry of Education, Culture, Sports, Science and Technology issued a nationwide directive to substitute online lectures of equivalent quality for in-person instruction and, if necessary, postpone certain lectures [11]. For institutions training future medical professionals, a guideline [12] was issued on 28 February and subsequently updated on 1 June, which permitted flexible adjustments to the schedule and facilities of clinical training without formal applications and encouraged alternative methods of medical education, including online clinical clerkships and assurance that, if necessary, students could obtain the necessary credits and practice hours to qualify for professional licensure examinations. 

However, minimal guidance was provided by the government to individual medical schools on how to conduct clinical clerkships during the COVID-19 pandemic, resulting in a diversity of approaches adopted by faculties in both lectures and clinical clerkships [13]. Faculty members utilized mailing lists and communication platforms such as Slack to share information and strategies among themselves [14]. As a result, many medical schools, such as the University of Tokyo [15] and Kyoto University [16], conducted lectures and clinical training online, and some even reported conducting on-campus training such as anatomy dissections via online platforms [17]. 

#### 3.1.2. Case Report: Osaka University

At Osaka University Medical School, all clinical clerkships and lectures were conducted in-person until the onset of spring break in March. However, upon the commencement of the new academic term in April, all clinical clerkships were suspended and replaced by online lectures utilizing the Zoom or Webex platforms. However, certain departments were unable to fully adapt to the online format, and instead provided students with reports and other materials for independent study. All planned clinical rounds at overseas or external medical facilities were also canceled, and written examinations were either postponed or replaced by written assignments.

Following the lifting of the state of emergency on 6 May, preparations were made for the resumption of on-site clinical training. As a result, clinical clerkships for fifth- and sixth-year medical students were resumed at Osaka University Hospital on 8 June, and medical students engaged in laboratory research were permitted to re-enter university facilities on 5 June. This resumption schedule was relatively early compared to other urban-based Japanese medical schools. In preparation, faculty members provided students with an online lecture and examination on standard precautions and infection control, entitled “Standard Conducts for Medical Staff”, as well as requiring students to monitor their health status, including body temperature and other basic symptoms, for at least two weeks prior to the reopening of the facility. Clinical training at this point was a hybrid of online and on-site instruction, varying by department, with training conducted primarily online, only in inpatient wards, or exclusively utilizing educational simulators. For lower-level classes, lectures were conducted online, but written examinations were conducted in dispersed rooms, implementing hand hygiene and maintaining social distancing.

In September, following the summer break, all clinical clerkships were fully resumed at the teaching hospitals. Students prior to the clinical clerkships were also granted access to university facilities for curriculum elements that were difficult to conduct online, such as anatomy practice and biochemistry experiments. For classes that emphasized student interactions, some students attended in-person while others participated remotely. Students were also instructed to continue monitoring their health status on a daily basis throughout the entire period.

As the number of COVID-19 cases began to rise in the autumn and winter, guidelines [18] and codes of conduct [19] for the students were revised and disseminated accordingly. The Objective Structured Clinical Examination (OSCE) schedule during this period for the fourth-year students in Osaka University was conducted as scheduled under sufficient infection control precautions. 

In anticipation of potential suspensions of clinical trainings due to increasing infections, alternative plans were devised, particularly for overseas training and community medicine training.

### 3.2. The United Kingdom of Great Britain and Northern Ireland (UK)

The UK experienced its first wave of positive COVID-19 cases in March, and also experienced another outbreak from November to December [20]. In March, the government conducted a nationwide lockdown under the slogan “stay at home, protect the NHS, save lives”. Educational institutions, including universities, were closed during this period.

During the pandemic, many medical schools adapted their curriculum in accordance with the guidelines issued by Public Health England (PHE) and the Medical School Council (MSC). On 10 January 2020, the PHE published “COVID-19: infection prevention and control guidance” [21], which provided essential information for infection control, including risk assessment for the mode of transmission, hygiene techniques, personal protective equipment (PPE), face masks and effective social distancing.

In consultation with the government and the General Medical Council (GMC), MSC also issued “Advice from Medical Schools Council to UK Medical Schools on actions surrounding COVID-19” [22], which prioritized higher-grade students as opportunities for clinical training became scarce. Lower-grade students were instructed to conduct lectures that were planned for later, and it was recommended the Objective Structured Clinical Examination (OSCE) be postponed.

The MSC issued the “Statement on clinical placements”, initially on 1 May [23] and later updated it on 3 July [24], which emphasized the necessity for medical students to observe and participate in clinical sessions, an experience that cannot be replicated online. It also designated medical students as “essential workers” for the National Healthcare Service (NHS) in the future and designated clinical training for medical students as “essential work”. This emphasized the importance of bringing medical students into practice whenever possible, considering the following three points: (1) the ability of the placement provider to safely supervise students on placement; (2) the availability of PPE within different sites; and (3) government advice on social distancing and travel. Students were also advised to participate in medical treatment as much as possible within their capacity, rather than solely observing. Simulation practice was provided as an alternative when considering the potential hazards posed by COVID-19. MSC also prioritized clinical training opportunities for final-year students, followed by senior-year students.

This statement also hinted at the possibility for medical students to participate in the response to COVID-19 as members of the NHS. The guidelines also discussed the re-evaluation of Electives and Student Selected Components, in order to compensate for lost training, and provided information to assist medical students in obtaining clinical experience and medical knowledge and skills in a manner as closely to normal as possible. The second version of the statement [24] provided information on the use of skill labs and simulation training for students prior to clinical training. GMC published “Supporting the COVID-19 response: Guidance regarding Medical Education & Training” [25] in March, which provided guidance for trainee participation in the NHS during emergencies. The MSC also published guidance for students involved in specific medical procedures as NHS volunteers [26].

Despite the issuance of numerous guidelines on medical education in the UK, the response to COVID-19 varied among universities, such as in regard to the administration of final-year examinations (OSCE and other written examinations) [27]. For example, Imperial College London [3] was among the first medical schools to provide fully remote medical education. It also established a format for students to participate in volunteer programs in hospitals and GP practices, supporting medical professionals on the frontlines. 

### 3.3. United States of America (USA)

#### 3.3.1. General Policies

After confirming the first case of COVID-19 on 21 January 2020 in Seattle, the virus rapidly spread across the United States. In response, the U.S. Department of Education and various organizations have issued guidelines and information on how to handle COVID-19 in educational settings. The Center for Disease Control and Prevention (CDC) has issued guidelines for K-12 schools to implement adequate preventive measures and protocols when a student’s health deteriorates [28]. The resumption of schools was to be decided based on the outbreak situation within the region, requirements by state and local health officials and the preparedness of the school (with regard to sufficient human resources and prevention schemes). For higher education institutions, as the risk of infection was dependent upon various factors, such as social life on and off campus, the guidelines required the categorization of student activities according to the risk level of infection and the resumption of education with sufficient preventive measures [29]. 

Guidelines for medical education have been developed by the American Medical Association (AMA) and the Association for American Medical Colleges (AAMC). In March, when the pandemic was spreading rapidly, the AAMC recommended postponing clinical practice [30]. However, as the shortage of medical staff became increasingly apparent, states began to consider including medical students in the workforce [31], with permission granted under the condition that participation adhered to several principles and guidelines, such as the necessity in the medical facilities and that participation was completely voluntary [32,33]. In April, considering that some medical students were participating in volunteer work and part-time jobs outside of their medical school curriculum, the AAMC issued regulations for Medical Students in Volunteer and Paid Clinical Work, outlining the factors that governments and medical schools should consider when permitting students in such off-campus activities, and stating that the responsibility of the students shifted to the organization sponsoring the students’ clinical work [34]. 

The AAMC continued to update its guidelines for clinical training. In April, it endorsed conducting clinical practice in communities that showed no trend of the spread of COVID-19 and where adequate personal protective equipment (PPE) and COVID-19 testing could be conducted [35]. This was to be held under strict safety measures and in accordance with the educational principles of each medical school. In May, the Coalition for Physician Accountability released a guideline on the residency cycle [36], and checklists for the resumption of clinical training were issued in July [37]. 

By August, as more information about COVID-19, including its diagnosis, prevention and standard precaution measures, became available and the shortage of future medical professionals due to the ongoing pandemic became more pressing, the importance of resuming clinical practice increased. Additionally, including medical students in the workforce as healthcare workers was permitted under the principle of complete voluntary participation and in an adequate environment with PPE, testing and supervising physicians [38]. 

The AMA first issued guidelines for infection control for residents and fellow physicians. In regard to medical education, in addition to the content of the guidelines released in May by the AAMC, two documents were released outlining considerations for early graduation [39] and evaluation criteria for adjusting the educational curriculum and methods due to the spread of infection [40].

#### 3.3.2. Case Report: University of California, San Francisco

The University of California, San Francisco (UCSF), began providing information on its response to COVID-19 in regard to medical education on 24 March [41], when the outbreak became prominent in the USA. The main principles were to ensure that students acquire necessary medical skills and continue their studies, while also adjusting the educational curriculum to lower the risk of infection for students and faculty. 

In April, specific guidelines [42] were released, including the implementation of evidence-based infection control measures and a policy to provide clinical practice and face-to-face classes whenever possible. Priorities were set for the training system, with priority given to fourth-year students whose curriculum consisted only of clinical practice, and then to third-year students, who had the residency matching process upcoming. Clinical practice was postponed until adequate personal protective equipment, patients, or supervisors could be provided and alternative measures were considered in the meantime. These measures were applied to students with diseases that may lead to severe conditions if co-occurring with COVID-19 infection.

The guidelines were amended in July, allowing students to conduct clinical practice when there were sufficient patients, PPE and supervisors. Allocation of students to other facilities was also considered when necessary. For lectures, it was decided that lectures consisting of more than 10 students were to be held online, but small group classes (particularly in physical diagnosis and simulation trainings), sensitive topics, preceptorships and other classes which would need face-to-face dialogue were to be held with all members coming to campus less than once a week on average. Students were not to have any contact with anyone other than specified members of the class in order to facilitate contact tracing when necessary. Public Harm Reduction Strategies (physical distancing and masking in public, universal hand hygiene and remaining at home if new symptoms develop) and the use of PPE, daily health monitoring using a physical condition management application and PCR testing once a week before returning to university grounds were mandated.

#### 3.3.3. Case Report: Harvard Medical School

Harvard University recommended remote access until June 2021, and campus entry was restricted to authorized staff and students only, requiring advance training for COVID-19 prevention for each individual and laboratory prior to entry [43]. In addition to basic infection control measures such as hand hygiene and mask wearing, a health management application was recommended to keep track of daily health status, and COVID-19 testing standards were adopted according to the outbreak risk. 

Harvard Medical School [44] conducted lectures in a hybrid format with online access as its principle. The lectures specifically emphasizing the importance of face-to-face interactions included physical examination in the Pathways Practice of Medicine course and the HST Introduction to Clinical Medicine course, as well as Objective Structured Clinical Exams (OSCEs) and hands-on laboratory exercises. Many laboratories also opened their doors to provide research opportunities. These on-site lectures, research and training sessions were requested to be conducted following institutional guidelines and public health developments. Contact points were also set up for those who were not feeling well. The faculty requested program leaders to inform students and staff frequently about each program’s essential information, such as the starting date, financial support, visa, travel and dormitory requirements. The primary goal of resuming face-to-face classes was set for January. 

### 3.4. Australia

After the initial detection of COVID-19 in Australia on 25 January 2020, the country saw a surge in cases during March and April, as well as a resurgence during July and August. The government initially conducted border control and national lockdowns to prevent the outbreak [45]. The Australian Government Department of Education, Skills and Employment emphasized the need for online education during the pandemic, while also affirming the efficacy of in-person education [46]. Decisions regarding education were delegated to the State and Territory Governments and non-governmental sector authorities. 

Concomitantly, a guideline for clinical practice was jointly issued by the Australian Government Department of Education, Skills and Employment and the Department of Health, recognizing the importance of clinical education in the development of medical professionals and the future Australian healthcare system. This guideline outlined eight principles to be followed, and required students to comply with government- and instructor-provided instructions, including social distancing and travel restrictions, to curb the spread of infection.

In response to such guidelines, several experiences by medical students have been reported, including Melbourne Medical School [47] and the University of Queensland [48,49]. Although on-site clinical training was recommended, some clinical trainings have been implemented using the hybrid online and on-site approach.

## 4. Discussion

The objective of this study was to analyze the key changes in medical education systems during the COVID-19 pandemic. While each country implemented unique strategies, a common goal among all nations was to bring medical students back to live education in a timely and safe manner. All countries evaluated the outbreak situation and determined when and to what extent students could return to clinical settings.

However, there were variations in the level of involvement of medical students and faculty in the treatment of COVID-19 patients across different countries. While some nations, such as the UK, allowed students to work as part of the medical staff to combat COVID-19 [50], others, such as Japan, adopted more cautious measures [13] to safeguard the well-being of both medical students and patients. Hence, as countries such as the UK and USA promoted the opportunities for medical students to experience encounters with COVID-19 patients, countries such as Japan restricted such opportunities. Each country should support medical students to secure optimal educational opportunities for each student albeit with the provision of comprehensive social and health support [50,51]. 

This may have led to the variation in the number of publications involving medical students. The number of publications regarding the experiences of COVID-19 from medical students from Japan were lacking compared to students from the USA and UK [52,53]. Although this may have been due to clinical inexperience during the primary period of the pandemic, the lessons and the legacy of all experiences of the pandemic should be recorded, especially to prepare for similar circumstances in the future. Although the pandemic reduced educational and research opportunities for students and trainees [54], innovative educational tools developed to reduce viral exposure could be useful as new educational opportunities [55,56]. Online training programs can be utilized anywhere in the world where the internet can be accessed, and recordings of the program contents can be accessed on-demand. As such, online training anywhere, anytime, for free may reduce the educational inequalities between various regions and postgraduate education, in which time restrictions hinder the trainees’ visits to on-site training sites [57]. However, as all countries have emphasized throughout their policies, the importance of on-site, live training should be reaffirmed, and opportunities to experience live training should be made available as much as possible.

Our review has several limitations. Firstly, our study is restricted to the education systems of high-income countries. In addition to the fact that the impact of COVID-19 varied between countries [58], prior to the onset of COVID-19, these countries were perceived to have a robust healthcare infrastructure in comparison to other nations. Consequently, this review only provides an insight into a limited aspect of medical education worldwide. As the COVID-19 pandemic is a global phenomenon, further research is required to evaluate its impact on medical education in other countries. Additionally, our study indicates that medical schools adopted varying measures during the pandemic, even within the same country. All nations included in our study granted some degree of flexibility in the curriculum of each medical school. This necessitated faculty members to improvise on their curriculum in accordance with the outbreak scenario within the region, but the extent of medical schools’ adaptation differed among countries. From our review, information from medical schools in the UK and Australia were mostly consistent with the general national guidelines; hence, case reports of these countries were not presented and were only mentioned throughout the general nationwide review process. However, as noted in Japan, the guidance provided by the government has been minimal, and countries such as Japan are expected to have a variety in how each medical school amended their educational curriculum. In order to comprehensively evaluate COVID-19’s overall impact on the medical education system, nationwide studies should be conducted to examine the curriculum of each medical school in detail.

Secondly, we observed a significant discrepancy in the number of references available for citation. Although case reports in the UK, USA and Australia could be found in several databases, resources for medical education in Japan were scarce [52], particularly in regard to references in English [53]. Furthermore, upon researching these references in 2022, some were no longer accessible. This highlights the significance of narrative case reports of medical education, particularly but not exclusively for Japan. Medical students and faculty members should be encouraged to present their cases in academic journals, as such experiences would promote further research not limited to the COVID-19 pandemic [59].

## 5. Conclusions

In conclusion, while the principle of conducting clinical training on-site remains paramount, countries have adapted novel methods to provide the best education possible during the pandemic. However, as the opportunities for the students varied between each country, the educational experiences throughout the primary period of the pandemic may have varied between nations. It is imperative that records of such training are recorded so that lessons learned during COVID-19 can inform potential future global crises.

## Data Availability

Data in this study are presented in the text; however, if the link to the references does not work, a copy of the references may be distributed by the authors upon reasonable request.
